# Investigating Viewership of Season 3 of “13 Reasons Why” and the Mental Wellness of Adolescents: Partially Randomized Preference Trial

**DOI:** 10.2196/25782

**Published:** 2021-09-15

**Authors:** Yalda T Uhls, Laurel Felt, Ellen Wartella, Andrew Sanders

**Affiliations:** 1 University of California, Los Angeles Los Angeles, CA United States; 2 University of Southern California Los Angeles, CA United States; 3 School of Communication Northwestern University Evanston, IL United States

**Keywords:** media, adolescence, mental health, narrative, 13 Reasons Why, conversation, television, depression, sexual assault

## Abstract

**Background:**

A conflicting body of research suggests that additional investigation is needed to understand how globally watched television shows featuring social and mental health issues, such as *13 Reasons Why*, might affect adolescents’ mental wellness.

**Objective:**

This study aims to investigate adolescents’ viewership of the third season of the Netflix drama *13 Reasons Why* (13RW-3) and their engagement with show-related content, paying special attention to mental health outcomes and conversational partners.

**Methods:**

A panel-based research platform operated by the National Opinion Research Center at the University of Chicago recruited 157 adolescents aged 13 to 17 years from its nationally representative pool of participants. Participants answered questions about how they discussed and learned about social and mental health issues portrayed in 13RW-3 (eg, masculine role pressure and sexual assault) and questions about mental wellness (eg, mental health self-efficacy and depression). After the participants completed the survey (T1), they were directed to either watch 13RW-3 as it aired for the first time (intervention group) or *not* watch 13RW-3 (control group). Approximately one month later (T2), all the participants were asked to complete the postsurvey. Additional survey questions about conversational partners, information seeking because of watching the show, and use of show-related content were included in the intervention postsurvey.

**Results:**

Our sample (N=157) was 52.2% (n=82) female and 54.8% (n=86) White, with a mean age of 14.99 (SD 1.4) years. At T2, viewers of 13RW-3 spoke about suicide significantly more frequently than nonviewers (*P*=.007). From T1 to T2, viewers increasingly discussed issues explored by 13RW-3 (*P*=.002), especially suicide, mental health, and bullying. Adolescent viewers were most likely to speak with friends, and parents were the second most commonly named. Two variables emerged as consistent moderators of conversational choices—having depressive symptoms and being impacted by sexual assault. There was no association between conversational frequency and information seeking around social and mental health issues, and neither mental health self-efficacy scores nor masculine role pressure scores significantly differed between viewers and nonviewers at T2.

**Conclusions:**

Viewing 13RW-3, a globally watched television show featuring social and mental health issues, led to adolescent conversations and information searches about topics explored by the show. Depressive symptoms and the impact of sexual assault moderated several relationships, guiding participants toward engaging with certain issues and seeking out specific conversational partners. As 13RW-3’s stories drove conversations—and story-driven conversations can raise awareness, reduce stigma, shift attitudes, normalize certain behaviors, and strengthen supportive relationships—potential wellness implications for television shows featuring social and mental health issues are considerable.

## Introduction

### Background

The first season of the Netflix drama *13 Reasons Why* (13RW-1), which examined a 17-year-old girl’s reasons for death by suicide, took the world by storm. It garnered unprecedented levels of engagement across social media, becoming the *most tweeted-about show ever* in the history of streaming television [1]. It drew favorable reviews from critics and viewers (78% and 80%, respectively, on the Rotten Tomatoes website) and high-profile recognition, including an Emmy nomination, a Golden Globes nomination, and a National Association for the Advancement of Colored People Image Award win.

However, the subject matter of 13RW-1 also elicited speculation of possible harm to youth. It flouted several recommendations for suicide-themed stories, including the established injunction against graphically depicting suicidal acts and newer best practices of providing trigger warnings and recommending resource providers, such as Crisis Text Line or Rape, Abuse & Incest National Network. In doing so, some argued that Netflix put a target on its back and put young people at risk. Myriad public health experts and organizations published condemnations, warnings, and recommendations [2-4], whereas many researchers used diverse methods to study the impact of the show [5-16].

For example, Ayers et al [5] found higher than predicted suicide-related keyword search activity on Google, whereas Cooper et al [6] found higher than predicted admissions to a children’s hospital because of an intent to self-harm. Another study found a postexposure worsening in mood that was particularly pronounced among the most vulnerable adolescent viewers [7]. Other researchers found that, among adolescent viewers who had ever considered suicide, a minority reported increases in suicidal ideation, whereas more than twice as many reported decreases [8]. A large survey of young adults, adolescents, and parents across 4 countries reported positive outcomes from viewing 13RW-1, including higher rates of suicide awareness, empathy, information seeking, outreach, and interpersonal discussion [9].

This conflicting corpus suggests that more research is needed to understand how television shows like *13 Reasons Why* (13RW) might affect adolescents. For example, despite numerous studies about the program, investigators had not conducted an experiment in real time, assigning adolescent participants to watch the show during its initial airing. Few studies investigated how viewers navigated show-related content. Finally, hardly any studies explored whether or how the show indirectly affected vulnerable youth by activating conversations in their networks, despite decades of research documenting how shows that spark interpersonal discussion are more likely to promote behavioral change [17,18].

### Developmental Stage

Adolescence is a critical age for identity development, characterized by elevated social-affective engagement and goal flexibility [19]. Physiologically, it is also a time when the brain is most receptive to the environment [20]. The sheer number of hours that adolescents spend with screen media daily—7 hours and 22 minutes, not including the time spent using screens for school or homework [21]—demonstrates that media shapes adolescents’ lived experiences.

Since the emergence of COVID-19, the number of hours that youth spend with media has skyrocketed [22], whereas early data [23] and youths’ own testimonies [24,25] suggest that the pandemic’s immediate and long-term impacts on adolescent mental wellness may be considerable. Entertainment media can contribute to adolescents’ well-being by destigmatizing mental health issues, modeling help-seeking behavior, and helping vulnerable youth to feel less alone. Collectively, this suggests that media consumed during adolescence may be an especially powerful socializing agent and thus raises the stakes around global media franchises targeted to young people.

### This Study

#### Overview

This study was designed to investigate adolescents’ viewership of the third season of *13 Reasons Why* (13RW-3) and their engagement with show-related content, with special attention paid to mental health outcomes and conversational partners. The research design was a two-wave panel in which adolescents were aged 13-17 years, recruited from across the United States, and answered a survey at each wave. We developed the questions in our survey by taking into account the extensive body of literature documenting the positive relationships among viewing entertainment, talking about the content, and seeking relevant information [17,18]. We also considered the 13RW-3 storyline, which prominently featured masculine role pressure, sexual assault, and the journey toward healing. Finally, we considered other 13RW studies’ examination of viewers’ mental health outcomes [14-16,26-29].

Between time 1 (T1) and time 2 (T2), participants were directed to either watch 13RW-3 (as it was aired for the first time) or *not* watch it. Our primary aim was to discover whether or how watching 13RW-3 was related to shifts in knowledge, attitudes, and behavior around mental wellness. Our secondary aim was to identify variables that moderated the experiences of more vulnerable adolescents.

#### Season 3 Summary

Although 13RW-1 unpacked protagonist Hannah’s death by suicide and the second season of 13RW pursued legal accountability for this tragedy, 13RW-3 centered on sexual assault and bullying. The show’s third season explored characters’ home lives, implicitly suggesting that masculine role pressure (enacted via elders’ homophobia, objectification of women, and physical abuse) might have facilitated antagonists’ peer-to-peer violence. Sexual assault survivors also took space and demanded justice. Similar to 13RW-1, the season’s first episode revealed the death of a young person, serial rapist Bryce Walker. Subsequent episodes unraveled how that happened and who was to blame.

#### Hypotheses

We hypothesized that after watching 13RW-3, adolescent viewers would speak about social and mental health issues more frequently than nonviewers and they had at T1, score higher on mental health self-efficacy measures than nonviewers, score lower on masculine role pressure measures than nonviewers, and seek out information about social and mental health issues. We also linked conversation and information seeking, hypothesizing that viewers who spoke about 13RW-3 and show-related issues would seek out more information about social and mental health issues than viewers who did not report these conversations. In addition to interrogating these hypotheses, we explored the moderating effects of age, gender, race, sexual orientation, and experiences with suicidal ideation, depression, and sexual assault.

## Methods

### Recruitment

A panel-based research platform operated by the National Opinion Research Center (NORC) at the University of Chicago recruited adolescents aged 13 to 17 years from its nationally representative pool of participants. NORC solely retained participants’ personally identifiable information, submitting anonymized data to our research team (details on their recruitment practices are given in Multimedia Appendix 1).

### Intervention

#### Overview

The NORC first reached out to parents of eligible adolescents to obtain their consent for their children to participate in the study. Next, they invited adolescents of consenting parents or guardians to take the T1 survey. If an adolescent clicked “YES” in the invitation, the NORC system brought them to a screener question—whether they had watched any of 13RW-3. If they had not (a prerequisite for participation), they were brought to a webpage that used a language approved by the institutional review board to support informed consent (Multimedia Appendix 2). When the adolescents clicked “YES” to indicate consent, the system brought them to the T1 survey. After completing the T1 survey, the NORC system randomly assigned participants to either watch 13RW-3 (intervention group) or *not* watch 13RW-3 (control group). The T1 survey was open for 1 month, closing on September 20, 2019. During this time, 263 participants (130 assigned to the intervention group and 133 assigned to the control group) completed the T1 survey.

The T2 survey was launched on September 26, 2019 (approximately a month after Netflix released 13RW-3) and closed on November 18, 2019. During this time, 157 participants completed the full survey—83 members of the intervention group and 74 members of the control group, representing a 59.7% (157/263) retention rate from T1 to T2. This rate is normative for social science research with teen and young adult populations [30], nearly mirroring a recent study by Cantrell et al [31] that retained 63.45% (7756/12,223) of its 15- to 21-year-old sample from T1 to T2.

#### Protocol Adherence

**Figure 1:**
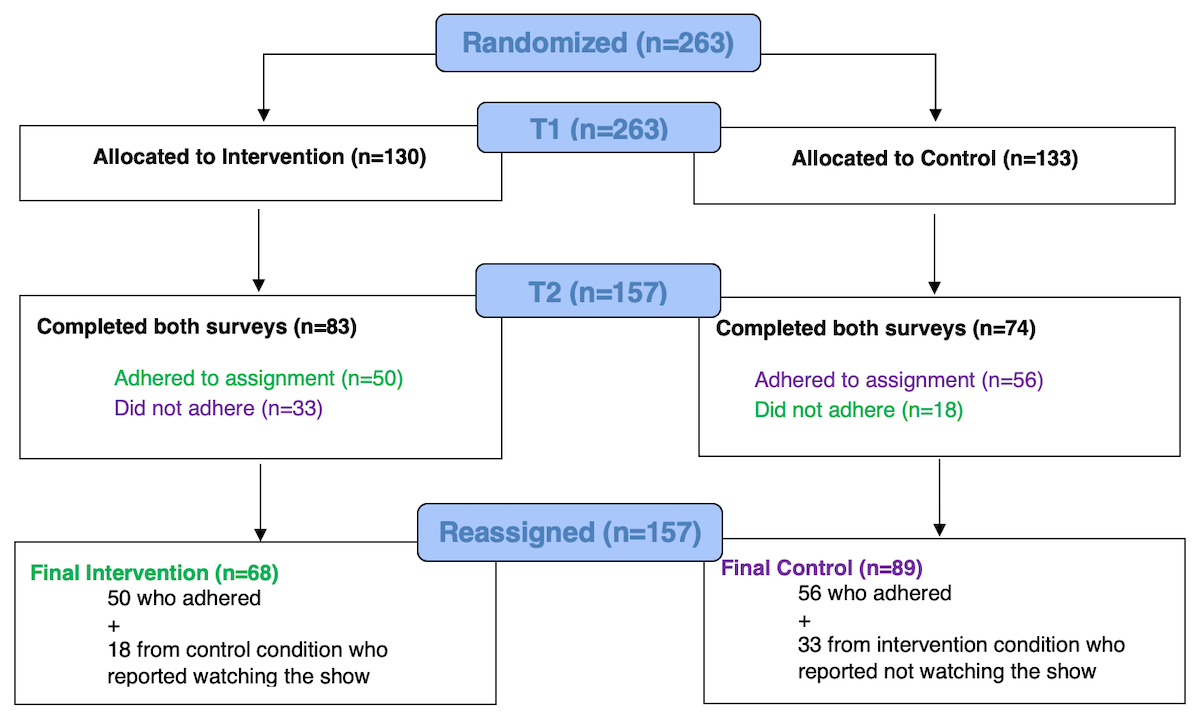
Participation flowchart from T1 to T2 to reassignment.

Despite the research team’s instructions to watch or not watch per random assignment, about one-third (51/157, 32.5%) of the sample disclosed in the T2 survey that they did not follow the protocol. Adolescent nonadherence is common across clinical and social science research. In this study, 51 participants did not adhere; 33 of the 83 participants randomly assigned to the intervention group did not watch the show, whereas 18 of the 74 participants randomly assigned to the control group watched the show (Figure 1).

Consequently, to maximize the statistical power, researchers shifted nonadherent participants to the group in which they actually participated so that their data would reflect their viewership [32]. After reassignment, the intervention group included 68 participants, with a quarter of these (18 participants) self-selecting into this condition from the control group; the control group included 89 participants, with approximately a third (33 participants) self-selecting into this condition from the intervention group (Figure 1). We used two-tailed independent sample *t* tests to compare whether the adherent and nonadherent participants differed on demographic data such as age, gender, race, sexual orientation, parental income, and geographical location and whether they had watched the previous seasons of the show. We found no differences between the 2 groups except for race (*χ^2^*_4_=11.5; *P*=.02), with Asian and mixed-race participants oversampling in nonadherence. We also ran independent *t* tests for all measures at T1 and found a significant difference for mental health self-efficacy (*t*_155_=–2.546; *P*=.01), with the nonadherent group scoring an average of 3.65 (SD 0.81) on this question versus 3.98 (SD 0.74) for the adherent group.

#### Protective Measures for At-Risk Adolescents

To support participants’ wellness, study participation was restricted to lower risk adolescents and all the participants were advised, “In the event that you disclose information about your possible intentions to do harm to yourself or others, NORC will provide information about your intentions to your parent(s) or legal guardian(s).” Therefore, if an adolescent answered in the T1 survey that they had seriously considered suicide, they were sent to a termination page and NORC personnel notified their parents. In addition to standard consent information, the survey offered a list of relevant resource providers (eg, Crisis Text Line) with the encouragement to “copy, screenshot, or print these resources now so that you can draw upon them at any time” (list given in Multimedia Appendix 3). The footer on every survey page at T1 and T2 also featured the following text: “If you are experiencing distress or discomfort, see this list of resources for help.” These procedures were approved by University of California, Los Angeles’s institutional review board.

### Measures

#### Overview

Approximately 6 weeks before the third season’s launch, 13RW-3 producers provided 3 members of the research team access to the show’s locked footage. This allowed researchers to craft a survey that asked about social and mental health issues central to 13RW-3 (eg, masculine role pressure, sexual assault, etc). Owing to space limitations, the described measures were found to be significant in the analysis. The full survey also included questions related to psychological and communication constructs (the full survey given in Multimedia Appendix 4).

#### Conversations Around Social and Mental Health Issues

A 7-item scale developed specifically for this investigation was used to assess conversational frequency around various social and mental health issues. Participants were asked how often they had spoken with their friends over the past 30 days about (1) *suicide* (*ie, considering and attempting*), (2) *mental health* (*ie, severe anxiety, anorexia, depression, etc*), (3) *bullying* (*ie, online and offline*), (4) *sexual assault* (*ie, inappropriate touching, lewd comments, rape, etc*), (5) *rigid gender stereotypes* (*ie, boys will be boys, girls are too emotional, etc*), (6) *substance abuse* (*ie, drugs, alcohol, etc*), and (7) *sexuality* (*ie, hookups, gender identity, sexual orientation, etc*). Response choices included 0=*not at all*, 1=*once a week*, 2=*a few times a week*, and 3=*every day or almost every day of the week.*

For participants in the intervention group only, the T2 survey included additional measures of conversation. One item asked whether participants discussed the 13RW-3 and show-related issues. The other item asked participants to select all of the people with whom they discussed 13RW-3 and issues related to the show after watching. Response choices included (1) *friends*, (2) *parents*, (3) *partner, boyfriend or girlfriend*, (4) *sibling*, (5) *teacher*, (6) *school counselor*, (7) *mental health professional or someone at a mental health resource hotline*, (8) *I did not discuss the show with anyone*, and (9) *other*.

#### Information Seeking

For participants in the intervention group only, 2 additional T2 survey questions were developed to assess whether viewers sought information about mental health issues either during or after watching the show. One item asked, “During or after watching the show, due to something you watched in the show, did you try to learn more about any of the following (through online search, asking an adult, etc.)?” Response choices included 10 items, such as (1) *suicide (considering and attempting)* and (2) *mental health (eg, severe anxiety, anorexia, depression, etc)*. Responses for each topic were tallied. The other item asked participants to select all of the show-affiliated crisis or informational resources that they visited. Response choices included 6 items (“www.13reasonswhytoolkit.org,” “13reasonswhy.info,” etc). Responses for each resource were tallied (the entire list of responses are given in Multimedia Appendix 4).

#### Masculine Role Pressure

A 6-item scale was developed to assess masculine role pressure, drawn from a survey used to inform Plan International’s *The State of Gender Equality for US Adolescents* report [33]. Participants were asked to indicate their agreement with questions such as “I know what makes a romantic relationship healthy” and “I know how to tell if my partner is uncomfortable in a sexual relationship” (the entire list of responses are given in Multimedia Appendix 4). Response choices across a 5-point scale ranged from 1 (*strongly disagree*) to 5 (*strongly agree*). Responses were averaged to create a composite score, with higher scores representing more masculine role pressure.

#### Sexual Assault

A 3-item scale assessed participants’ experiences with sexual assault, drawn from the 2017 Youth Risk Behavior Survey [34]. Participants were asked to indicate whether the following had occurred: “Have you ever been forced to have sexual intercourse when you did not want to?” “Have you ever been forced to do sexual things that you did not want to do? (Count such things as kissing, touching, or being physically forced to have sexual intercourse),” and “Are you close with anyone (friend, family member, romantic or sexual partner) who has ever forced someone else to do sexual things that the other person did not want to?” Response choices included 1=*Yes*, 2=*No*, and 3=*I don’t know*.

#### Depression

To assess participants’ level of depression, we used the Patient Health Questionnaire-2, a practical screening tool [35] that has been validated with adolescents [36]. Participants were asked how often they were bothered by each of the following symptoms during the past 7 days: (1) “Feeling down, depressed, irritable, or hopeless,” (2) “Little interest or pleasure in doing things.” Because of the complex role that sleep plays in adolescent depression [37], we added one item from the Severity Measure for Depression—Child Age 11-17 (adapted from Patient Health Questionnaire-9 modified for adolescents) [32] that asked how often participants experienced (3) “Trouble falling asleep, staying asleep, or sleeping too much.” Response choices included 1=*not at all*, 2=*several days*, 3=*more than half of the days*, and 4=*nearly every day.* Item scores were averaged to create a composite score, with higher scores representing more depressive symptoms.

#### Mental Wellness Self-Efficacy

A 3-item scale was developed to assess participants’ (1) mental health self-awareness (“I can recognize my own mental health-related ‘warning signs,’ or indicators that my own mental health may be poor”), (2) knowledge about mental health resources (“I know how to find helpful mental health-related information and/or professional support”), and (3) behavioral intention to access necessary mental health support (“If/whenever I need mental health-related information and/or professional support, I would reach out to get what I need”). Response choices across a 5-point scale ranged from 1 (*Strongly disagree*) to 5 (*Strongly agree*). Item scores for this scale were averaged to create a composite score, with higher scores representing higher mental wellness self-efficacy.

### Data Analysis

Descriptive statistics and frequencies were used to analyze the demographic data of the participants. A combination of statistical tests, including *t* tests, one-way analysis of variance (ANOVA), chi-square tests, and logistic regressions, were used to analyze the impact of the intervention. Difference scores were calculated between measures at the two time points (representing increases or decreases in measures from T1 to T2) by subtracting T1 data from T2 data (T2−T1). Posthoc power analyses were conducted using G*Power [38]. Sets of analyses primarily using *t* tests and one-way ANOVA were conducted independently to compare (1) intervention versus control groups and (2) the intervention group only. For the intervention versus control analyses, the power analysis of our 2-tailed *t* tests, measuring the differences between the experimental and control groups, resulted in a power of β=.802 (control: N=89; intervention: N=68; Cohen *d*=0.455; α=.05). The power analysis of our one-way ANOVA, measuring fixed effects omnibus tests between our 2 groups, resulted in a power of β=.968 (N=157; Cohen *f*=0.306; α=.05). For the intervention-only analyses, the power analysis of our 2-tailed *t* tests, measuring a difference from a constant, resulted in a power of β=.896 (N=68; Cohen *d*=0.396; α=.05). SPSS version 26 was used for all analyses.

## Results

### Participants

The final sample consisted of 157 participants—68 in the intervention group and 89 in the control group (demographics broken down by conditions are given in Table 1). The mean age of the sample was 14.99 years. The sample was fairly well balanced in terms of gender, with 82 females and 75 males. Most of the sample 54.8% (86/157) was White and non-Hispanic, 18.5% (29/157) identified as Hispanic, 17.2% (27/157) identified as Black, non-Hispanic, 6.4% (10/157) identified as mixed race, and 3.2% (5/157) identified as Asian, non-Hispanic.

**Table 1:** Demographics of participants.

Demographics			Intervention group (n=68)		Control group (n=89)
Age (years), mean (SD)			15.06 (1.35)		14.91 (1.49)
**Gender, n (%)**					
	Male	33 (49)		42 (47)	
	Female	35 (51)		47 (53)	
**Race, n (%)**					
	Combined White, non-Hispanic	35 (52)		51 (57)	
	Black, non-Hispanic	13 (19)		14 (16)	
	Hispanic	14 (21)		15 (17)	
	Mixed	4 (6)		6 (7)	
	Asian, non-Hispanic	2 (3)		3 (3)	
**Geographical location, n (%)**					
	Northeast	10 (15)		7 (8)	
	The Midwest	17 (25)		28 (32)	
	South	23 (34)		34 (38)	
	West	18 (27)		20 (23)	
Household income (US $), range			40,000-49,000		50,000-59,000
**Sexual orientation, n (%)**					
	Heterosexual	51 (75)		75 (84)	
	Bisexual	3 (4)		3 (3)	
	Asexual	2 (3)		2 (2)	
	Homosexual	0 (0)		3 (3)	
	Other or prefer not to say or do not know	12 (17)		6 (7)	

### Conversations Around Social and Mental Health Issues

At T2, viewers of 13RW-3 (mean difference [M_diff_] 0.368, SD 0.827) engaged in conversations around suicide significantly more frequently (*t*_124.580_=–2.735; *P*=.007; Cohen *d*=0.455) than nonviewers (M_diff_ 0.034, SD 0.651). In addition, compared with T1, at T2, the viewers engaged in significantly more conversations (M_diff_ 0.368, SD 0.827) about social and mental health issues (*t*_66_=3.243; *P*=.002; Cohen *d*=0.396), with the number of conversations about suicide (M_diff_ 0.367, SD 0.827), mental health (M_diff_ 0.324, SD 0.999), and bullying (M_diff_ 0.294, SD 0.915) increasing the most.

Two variables emerged as moderators of conversational frequency: depressive symptoms and knowing a survivor of sexual assault. Viewers with higher levels of depressive symptoms at T2 reported more frequent conversations about suicide from T1 to T2 than viewers with lower levels of depressive symptoms (*F*_8,59_=2.265; between-groups mean square [MS]=1.345; *P*=.04; Cohen *f*=0.554). Knowing a survivor of sexual assault moderated how often viewers spoke about bullying and sexuality, respectively, from T1 to T2. Viewers who knew a survivor of sexual assault reported more frequent conversations about bullying (*F*_1,57_=5.318; MS=3.813; *P*=.03; Cohen *f*=0.306) from T1 to T2 than viewers who did not. Viewers who knew a survivor of sexual assault spoke about sexuality less frequently from T1 to T2 (*F*_1,57_=9.985; MS=10.161; *P*=.003; Cohen *f*=0.419) than viewers who did not know a survivor of sexual assault.

### Mental Wellness Self-Efficacy and Masculine Role Pressure

After watching 13RW-3, the scores of adolescent viewers on mental wellness self-efficacy measures (M_diff_–0.152, SD 1.010) did not significantly differ (*t*_155_=0.746; *P*=.46) from nonviewers’ scores (M_diff_–0.041, SD 0.853). After watching 13RW-3, scores on masculine role pressure measures (M_diff_ 0.072, SD 0.613) did not significantly differ (*t*_151_=–0.134; *P*=.89) from nonviewers’ scores (M_diff_ 0.058, SD 0.625).

### Information Seeking

After watching 13RW-3, nearly all viewers (58/63, 92%) sought information on social and mental health issues. *Bullying* and *mental health* were the most frequently searched issues.

Once again, depressive symptoms and experiences with sexual assault emerged as moderating variables, influencing the extent to which viewers sought information. Viewers who reported little interest or pleasure in doing things sought information about suicide more than those who did not (B=0.806, SE 0.319; *df*=1; Exp (B)=2.238; *P*=.01; frequencies and significant moderators are given in Table 2). Viewers who reported having trouble with sleep sought more information about rigid gender stereotypes than those who did not report such sleep issues (B=0.663, SE 0.326; *df*=1; Exp (B)=1.940; *P*=.04). Viewers who self-identified as being forced to do sexual things they did not want to (kissing, touching, etc) sought more information about sexual assault than those who did not (Fisher exact test, *P*=.047). Viewers who reported being close to a survivor of sexual assault sought out more information about rigid gender stereotypes than those who did not report such a relationship (Fisher exact test, *P*=.03).

Approximately one-third (21/63, 33%) of viewers engaged with informative resources produced by or affiliated with 13RW (the entire list is given in Table 3).

**Table 2:** Information seeking by the intervention group and significant moderators.

Topic of information sought	Frequency (n=68), n (%)^a^	Significant moderators
Suicide (eg, considering and attempting)	10 (15)	Depression subscore: little interest or pleasure in doing things (B=0.806, SE 0.319; *df*=1; Exp (B)=2.238; *P*=.01)
Mental health (eg, severe anxiety, anorexia, and depression)	21 (31)	N/A^b^
Bullying (eg, web-based and face-to-face)	23 (33)	N/A
Sexual assault (eg, inappropriate touching, lewd comments, and rape)	16 (24)	Sexual assault subscore: being forced to do sexual things they did not want to (Fisher exact test, *P*=.047)
Rigid gender stereotypes (eg, boys will be boys)	11 (16)	Sexual assault subscore: Knowing a survivor (Fisher exact test, *P*=.03) Depression subscore: Trouble with sleep (B=0.663, SE 0.326; *df*=1; Exp (B)=1.940; *P*=.04)
Substance Abuse (ie, drugs, alcohol, etc)	13 (19)	N/A
Sexuality (eg, hookups, gender identity, and sexual orientation)	8 (12)	N/A
Abortions	8 (12)	N/A
Illegal immigration	9 (13)	N/A
Other (please specify)	2 (3)	N/A

^a^The question was “During or after watching the show, due to something you watched in the show, did you try to learn more about any of the following (through web-based search, asking an adult, etc)?” Frequency numbers represent those who checked this response item. Differences were tested by age, gender, race, sexual orientation, depression, and answers to questions about sexual assault.

^b^N/A: not applicable.

**Table 3:** Engaging with 13 Reasons Why-produced resources (intervention group).

Resource	Frequency (n=63), n (%)^a^
www.13reasonswhytoolkit.org	4 (6)
www.13reasonswhy.info	11 (16)
Discussion Guide on the 13 Reasons Why information webpage	3 (4)
Beyond The Reasons documentary on Netflix	14 (21)
Did not visit any 13 Reasons Why–related crisis or information resources	42 (62)

^a^The question was “Which crisis/informational resources did you visit or watch that are affiliated with the show?” Frequency numbers represent those who checked this response item.

### Conversational Partners

The vast majority of viewers (60/68, 88%) reported speaking about 13RW-3 and issues related to the show. Adolescent viewers most commonly chose friends (43/68, 63%) as their conversational partners, followed by parents (32/68, 47%); frequencies and significant moderators are given in Table 4).

**Table 4:** Conversational partners (intervention group).

Conversational partner	Frequency (n=68), n (%)^a^	Significant moderators
Friends	43 (63)	Sexual assault subscore: viewers who reported knowing a survivor spoke to their friends more than those who did not know a survivor (Fisher exact test, *P*=.047)
Parents	32 (47)	Depression subscore: Viewers who reported feeling down, depressed, irritable, or hopeless spoke to their parents less (B=–0.923, SE 0.373; *df*=1; Exp (B)=0.397; *P*=.01)
Partner or boyfriend or girlfriend	9 (13)	N/A^b^
Sibling	13 (19)	N/A
Other family members	7 (10)	Race or ethnicity: Black and mixed-race participants reported speaking more to other family members (*χ*^2^_4_=13.8; *P*=.008)
Teacher	5 (7)	N/A
School counselor	2 (3)	N/A
Mental health professional or someone at a mental health resource hotline	1 (2)^c^	N/A
No one	8 (12)	N/A

^a^The question was “With whom did you discuss 13 Reasons Why and issues related to the show after watching?” Frequency numbers represent those who checked this response item. Differences were tested by age, gender, race, sexual orientation, depression, and answers to questions about sexual assault.

^b^N/A: not applicable.

^c^This respondent reported feeling depressed nearly every day.

In addition to depressive symptoms and knowing a survivor of sexual assault, race or ethnicity also moderated the extent to which viewers spoke about 13RW-3 and show-related issues with certain conversational partners. Viewers who reported higher levels of feeling down, depressed, irritable, or hopeless at T2 spoke to their parents less (B=–0.923, SE 0.373; *df*=1; Exp (B)=0.397; *P*=.01) than those who reported lower levels of depressive symptoms. Viewers who knew a survivor of sexual assault spoke to their friends more than those who did not know a sexual assault survivor (Fisher exact test, *P*=.047). Viewers who identified their race or ethnicity as *Black, non-Hispanic* and *Mixed* spoke with *other family members* (ie, neither parents nor siblings) more than viewers who identified with other races or ethnicities (*χ^2^*_4_=13.8; *P*=.008).

## Discussion

### Principal Findings

As hypothesized, watching the show was associated with significantly more conversations about social and mental health issues. A vast majority (60/68, 88%) of viewers reported discussing the show and related issues. The potential wellness implications were significant. Story-driven conversations can raise awareness, reduce stigma, shift attitudes [39], normalize certain behaviors [40], and strengthen supportive relationships [41]. For example, in a study of a popular TV show with a public health storyline, viewers’ interpersonal discussions facilitated and amplified the show’s impact; when viewers discussed the show, they were more likely to demonstrate show-related shifts in knowledge, attitude, and behavior [42]. By supporting adolescents’ narrative-inspired conversations, parents, educators, and public health officials may reap these types of benefits.

As expected, adolescents most often reported *friends* as their conversational partner (43/68, 63%); more surprisingly, *parents* did not trail far behind. Almost half of the sample participants (32/68, 47%) chose to discuss the show and issues related to the show with parents, with no differences based on gender or age. Intrigued by this finding, we searched for other parent data. We found that 60% (36/60) of 13RW-3 viewers watched the show with a parent at least once, similar to a global study on 13RW-1 [9] wherein 39.1% (184/471) of its sample reported coviewing with a parent and 43% (123/286) of those who did not coview reported talking about the show with a parent. Collectively, these data present parents as important members of adolescents’ media ecologies, at least with respect to shows like 13RW. Despite adolescents’ engagement with individuation, a developmental imperative that has them turning away from parents and toward their peer group [43], adolescents seem to rely on parents’ support for processing this challenging content—even traditionally, harder-to-reach adolescents, such as males.

Supporting our hypothesis, nearly all viewers (58/63, 92%) tried to learn more about issues explored by the show, with *bullying* and *mental health* drawing the most searches. This high rate of engagement outstrips a recent research finding [44], in which approximately half of an adult sample sought more information about something they learned from a fictional movie or TV show. Approximately one-third (21/63, 33%) of viewers engaged with informative resources produced by or affiliated with 13RW. These findings suggest that providing credible, engaging resources is a worthwhile effort for content creators for reasons that are not simply performative.

### Additional Findings

We also aimed to identify moderator variables, particularly those that moderated the experiences of more vulnerable adolescent viewers. Our explorations of age, gender, race, and sexual orientation and relationships with suicidal ideation, depression, and sexual assault revealed that lived experience—rather than demographic characteristics—were most likely to significantly affect viewers’ decisions.

Depressive symptoms emerged as an important moderator. Two of our findings related to engaging with the topic of suicide, namely, viewers who reported higher levels of depression talked about suicide more frequently at T2 than those with lower levels of depression. In addition, viewers who reported that they had little interest or pleasure in doing things (a subscale of our depression measure) looked up information on suicide more frequently. It is important to note that the content of these conversations and searches is unknown. Although the popular press tended to frame similar findings from Ayers et al [5] as sinister, assuming or implying that suicide-related information seeking meant procuring strategies on how to end one’s life, this is not necessarily the case. Depressed youth might have sought information or broached a conversation about suicide to get help; for youth, talking about suicide can serve as a protective factor [45]. It is also possible that depressed adolescent viewers initiated conversations to reassure their support networks that, despite their affect, they were not contemplating death by suicide. Interestingly, teen suicide rates declined in 2019, the year in which the show came out [46].

We also found that viewers who reported feeling down or depressed spoke to their parents less than nondepressed peers. This is unsurprising, given the nature of depression, which commonly reduces individuals’ interest in social interaction. Depressed adolescents might have been particularly loath to speak with their parents, who may have been worried about or critical of their condition.

Being affected by sexual assault also moderated several relationships. Adolescent viewers who knew a survivor of sexual assault spoke about bullying more than their peers, whereas they talked about sexuality less. This might suggest viewers’ learning from 13RW-3, which framed sexual assault as oppression—a means for violent bullies to exert power—and not as intimacy. Owing to their knowledge of their friends’ experiences, these viewers might have been more receptive to such framing and/or more curious to learn more.

Indeed, viewers who knew survivors of sexual assault sought information about rigid gender stereotypes more than their peers. Again, this hints at learning from 13RW-3. Season 3 showed how rigid gender stereotypes, especially a set of male heterosexual stereotypes commonly known as toxic masculinity, shaped the home life of the show’s serial rapist. It is possible that, because of their friendship with sexual assault survivors, these viewers were particularly interested in discovering whether or how internalizing rigid gender stereotypes can lead to sexual assault. Similarly, viewers who reported having trouble with sleep (a subscale of our depression measure) sought more information about rigid gender stereotypes than those who did not report such sleep issues. This is consistent with international research that has identified an association between traditional gender role attitudes and poor mental health outcomes (eg, depression and suicidal ideation) [47-49]. Finally, viewers who reported surviving sexual assault sought more information about it than their peers. Owing to their lived experience, these viewers likely were more motivated to make sense of this scourge and work toward healing. Of note, healing from sexual assault was a significant storyline in 13RW-3.

Finally, viewers who identified their race or ethnicity as *Black, non-Hispanic* and *Mixed* spoke with *other family members* (ie, neither parents nor siblings) more than viewers who identified with other races or ethnicities. This aligns with the established research that has found that “the extended kin network is a more salient structure for Black families than it is for White families” [50] and a newer research finding that African Americans and Black Caribbeans give and receive support from extended networks (eg, fictive kin and fellow congregants) more than non-Hispanic White people [51].

### Limitations

By design, study participation was limited to adolescents who reported they had never seriously considered suicide; as such, we cannot answer whether or how 13RW-3 impacted suicidal youth. As we only recruited adolescents across the United States, our results are limited to this population.

We did not measure the valence of adolescents’ conversations and/or information seeking and thus cannot definitively claim that these actions were positive. Depending on how the conversations unfolded, the more vulnerable youth may have suffered or benefited. Our modest qualitative data suggest, however, that viewers embraced a prosocial message (eg, “The show made me think about the things that teens go through [sic] and then on top of that all the bullying and family problems. I can now say I am much more careful about the things I say. I was made aware of the language I use on a day to day basis”).

Other limitations emerged in process. Our sample size, although considerable, could have been larger. Some participants did not adhere to the randomized conditions, making the study a partially randomized preference trial rather than a randomized controlled trial. Adolescent nonadherence is common across clinical and social science research. For example, according to the Children’s Hospital of Philadelphia [52], adolescent transplant recipients disregard the protocol at a 60% higher rate than adults, although such behavior risks their lives. We found a significant difference between adherents and nonadherents on the mental health self-efficacy measure, which may have influenced our results. In addition, the 18 nonadherents originally assigned to the control condition did not receive T2 questions about 13RW-3.

Finally, some of our measures were not validated, and this may have affected our outcomes. To assess both mental health self-efficacy and masculine role pressure, we used the unvalidated scales that we developed for this study. Both outcomes also may suggest insufficient *dosage*—that is, perhaps the storyline of 13RW-3 met the threshold to raise consciousness and catalyze discussion around social and mental health issues but did not deliver (enough) content to impact specific constructs.

### Comparison With Previous Work

The neutral and positive outcomes documented in our study align with a growing collection of studies that have found salubrious effects associated with watching 13RW [9,10,14,26-29]. These effects include lower depressive symptoms [27], decline in incidences of suicidal ideation and self-harm [14], decreases in stigmatized beliefs about suicide [27], increased sense of mental illness being socially acceptable [26], increased knowledge of suicide risk factors [28], engagement in prosocial mental health–related behaviors [26], and increased likelihood of expressing interest in helping a suicidal person [14]. It also strengthens another group of methodologically driven studies that problematize claims of 13RW viewership, leading to harm [11,29]. Of note, a reanalysis [11] of the data presented by Bridge et al [29] repudiated (widely reported) claims that suicide rates increased in the months following the debut of 13RW.

### Future Research

Our findings suggest that globally watched television shows featuring social and mental health issues drive conversation and that social media are regarded as a promising avenue to support adolescents’ well-being [53-55]. Thus, we are currently exploring whether second-screen content (ie, content appearing on a phone, tablet, or laptop that does not simultaneously exhibit a television show) can amplify the impacts of a narrative by facilitating conversations and information seeking around the topics raised within the show.

As noted, we do not know the content of adolescents’ conversations. Future research should examine the impact and valence of narrative-inspired conversations on vulnerable youth. For example, scornful conversations might deliver a boomerang effect, intensifying mental health stigma. Appreciative conversations might enrich vulnerable adolescents’ social context, normalizing challenges around mental wellness and improving peers’ abilities to identify warning signs. Future research might also investigate narrative-inspired information seeking. Are adolescents more likely to seek information about topics featured in popular media than adults? Do narratives like 13RW drive more information seeking than other types of narratives?

Understanding whether and how parents’ media choices impact adolescents—for example, whether subscribing to streaming services such as Netflix or whether enforcing media rules is associated with adolescents’ mental wellness—would be another fruitful arena of investigation. Similarly, researchers could query whether and how adolescents’ media choices affect parents (known as the *child effect* [56]). A recent study found that children actively mediate their parents’ television use [57], a phenomenon attested by our own study and a global study on 13RW-1 [9]. Therefore, it would be interesting to examine how parent-child engagement with adolescent-selected media might affect the parent-child relationship and parenting practices.

Finally, it is important for future research to examine the more vulnerable minority—youth with a history of suicidal ideation—and whether and how narrative affects their ideation.

### Implications and Conclusions

Our findings should have implications for diverse stakeholders, including entertainment content creators, studios, public health experts, parents, and adolescents seeking to support adolescent mental health. Previous research has documented popular film and television’s tendency to stigmatize and trivialize mental health [58], stoking some creators’ resolve to correct mental health’s representation or misrepresentation. This study suggests that such efforts do not just improve the optics; they can facilitate meaningful outcomes in viewers’ lives. It complements recently released data on narratives’ capacity to shift viewers’ mindsets, “which can transform the way Americans think about health and influence policy” [44]. Therefore, executives can use this research to emphasize how stories matter and further inspire entertainment content creators’ efforts to enrich representation. As their content can drive conversations and information seeking, studios could also create resources that amplify these outcomes, including mini documentaries like *Beyond the Reasons* (the most used resource in our sample) and omnichannel promotion of companion content. Companion content might include conversational toolkits created by public health experts, designed to support the two most common types of dialogues: peer-to-peer and adolescent-parent. Clinicians and advocacy organizations can leverage the insight that adolescents who are most sensitive to this content are those who cope with depressive symptoms and/or are affected by sexual assault. First, they can seek to proactively identify adolescents who meet these criteria. Second, they can tailor programming to account for their lived experiences.

Most importantly, this investigation suggests that when all stakeholders work together well in advance of a global franchise’s release, they can better harness the content’s potential to enrich adolescent wellness. In fact, several entertainment outlets (eg, MTV, Comedy Central, VH1, and Paramount Plus) recently committed to representing mental health with greater frequency, accuracy, and empathy [59]. Indeed, this study’s lead author is speaking to this group about how the findings can enhance the resources for content creators.

## Appendix

Multimedia Appendix 1 Head of household consent email.

Multimedia Appendix 2 Adolescent consent.

Multimedia Appendix 3 Resources for parents, guardians, or heads of household.

Multimedia Appendix 4 Postsurvey intervention.

## Abbreviations

13RW: 13 Reasons Why
13RW-1: 13 Reasons Why, Season 1
13RW-3: 13 Reasons Why, Season 3
ANOVA: analysis of variance
MS: between-groups mean square
NORC: National Opinion Research Center
