# Supplementary material for: Investigating Viewership of Season 3 of “13 Reasons Why” and the Mental Wellness of Adolescents: Partially Randomized Preference Trial
Source: JMIR Ment Health. 2021 Sep 15;8(9):e25782. doi: 10.2196/25782 (PMC8482170; doi:10.2196/25782)
Supplement: Multimedia Appendix 1 [file mental_v8i9e25782_app1.docx]

**APPENDIX A. Head of Household Consent Email**

You are asked to allow [CHILD’S NAME] to participate in a research study. Below is more information about the study.

**PURPOSE OF THE STUDY**

We are looking to understand how adolescents respond to certain topics in the TV show *13 Reasons Why*. This show has been extremely popular with teens. Some concerned advocates have speculated that vulnerable adolescents may derive harm from watching the show, perhaps via increased suicidal ideation and/or depression, while others have suggested that watching the show led to positive conversations and increased empathy.

As researchers, we are interested in understanding how to maximize the positive benefits of the show for young viewers while minimizing the negative effects. This research seeks to examine this question.

**WHAT WE WILL ASK OF YOUR CHILD**

If you agree to allow [CHILD’S NAME] to participate in this study, we would randomly assign them to an experimental or control group. Each child will then complete the following:

1. Complete a 10 minute survey online.
2. If assigned to the experimental group, we will ask your child to watch the entire third season (13 one hour episodes) of *13 Reasons Why*, over a period of one month. If assigned to the control group, we will ask them to continue their media diet as usual with no additional content to watch.
3. In one month, complete another 10 minute survey.

**POTENTIAL RISKS AND DISCOMFORTS**

Season three content is primarily concerned with sexual harassment, homophobia and shame and toxic masculinity. Some material may be challenging and sensitive viewers may not enjoy it.

**POTENTIAL BENEFITS TO SUBJECTS AND/OR TO SOCIETY**

Your child’s participation in this research may help us to better understand how to best support adolescent mental health watching a popular television show.

**INCENTIVE FOR PARTICIPATION**

You will receive 2,000 Ameripoints for providing your consent for us to contact [CHILD’S NAME]. [CHILD’S NAME] will receive 5,000 Ameripoints for completing the first survey, 5,000 Ameripoints for completing the second survey, and 10,000 additional Ameripoints for watching all thirteen episodes of the show.

Do we have your permission to contact [CHILD’S NAME] to participate in this study?

1. Yes – generates email invitation to teen
2. No – makes household
