# Supplementary material for: Investigating Viewership of Season 3 of “13 Reasons Why” and the Mental Wellness of Adolescents: Partially Randomized Preference Trial
Source: JMIR Ment Health. 2021 Sep 15;8(9):e25782. doi: 10.2196/25782 (PMC8482170; doi:10.2196/25782)
Supplement: Multimedia Appendix 2 [file mental_v8i9e25782_app2.docx]

**APPENDIX B. ADOLESCENT CONSENT**

[DISPLAY – WINTRO_1]

Thank you for agreeing to participate in our new AmeriSpeak survey! To thank you for sharing your opinions, we will give you a reward of [SHOW IF NOM_TYPE=1: [INCENTWCOMMA] AmeriPoints] [SHOW IF NOM_TYPE=2: a $10 Amazon gift card] after completing this survey. As always, your answers are confidential.

*Please use the “Continue” and “Previous” buttons to navigate between the questions within the questionnaire. Do not use your browser buttons.*

[DISPLAY1]

You are asked to participate in a research study conducted by Dr. Yalda T. Uhls, a researcher at UCLA. You were selected as a possible participant in this study because of your age (13 to 17 years old). Your participation in this research study is voluntary.

[SPACE]

<u>PURPOSE OF THE STUDY</u>

We are looking to understand how adolescents respond to certain topics in the TV show 13 Reasons Why. As researchers, we are interested in understanding how to maximize the positive benefits of show for young viewers while minimizing the negative effects. This research seeks to examine this question.

[SPACE]

<u>PROCEDURES</u>

If you agree to participate in this study, below is what we would ask you to:

- Complete this survey
- [SHOW IF DOV_EXP=1] You will need to watch the entire third season (13, one hour episodes) of 13 Reasons Why, over a period of one month. You will be compensated with an additional [SHOW IF NOM_TYPE=1: [INCENTWCOMMA] AmeriPoints; SHOW IF NOM_TYPE=2: $10 Amazon gift card]!
- [SHOW IF DOV_EXP=2] We ask that you do not watch the third season of 13 Reasons Why for a period of one month. You will be compensated with an additional [SHOW IF NOM_TYPE=1: [INCENTWCOMMA] AmeriPoints; SHOW IF NOM_TYPE=2: $10 Amazon gift card]!
- You will then be asked to complete a follow-up survey. Once completed you’ll receive another [SHOW IF NOM_TYPE=1: [INCENTWCOMMA] AmeriPoints; SHOW IF NOM_TYPE=2: $10 Amazon gift card], for a total of [SHOW IF NOM_TYPE=1: 30,000 AmeriPoints; SHOW IF NOM_TYPE=2: $30]!

[SHOW IF DOV_EXP=1]

[DISPLAY2]

<u>POTENTIAL RISKS AND DISCOMFORTS</u>

Season three content is primarily concerned with sexual harassment, homophobia and shame and toxic masculinity. Teen dramas commonly engage with tough topics, like bullying and suicide. The first two seasons of the show *13 Reasons Why* presented topics/issues related to suicide. While suicide is not the focus of season three, it was the focus of previous seasons and allusions to it may make you feel uncomfortable. There is some risk of psychological or emotional stress or discomfort from watching shows that explore these topics and/or from answering related questions. You can skip any questions you do not wish to answer or exit either survey at any point. You can stop watching an episode at any point or stop watching the assigned show altogether.

[SPACE]

<u>POTENTIAL BENEFITS TO SUBJECTS AND/OR TO SOCIETY</u>

We hope that this research will help us to better understand how teen dramas may affect diverse adolescents; such insight can help storytellers to craft content that supports adolescents’ wellbeing.

[DISPLAY_HELP]

This survey will ask you some difficult questions on topics that may cause distress including, suicide and sexual harassment. If you experience distress, know of anyone experiencing distress, or simply want to learn more about these topics, you can access help and information here:

PROGRAMMING NOTE: TEXT COLOR - RED

- **stopbullying.gov**
- **crisistextline.org**
- **suicidepreventionlifeline.org**
- **rainn.org**
- **1-800-273-TALK (8255)**
- **Text HOME to 741741**

We encourage you to copy, screenshot, or print these resources now so that you can draw upon them at any time.

[DISPLAY_CONF]

<u>CONFIDENTIALITY</u>

This survey is hosted by NORC at the University of Chicago (www.norc.org). As for all AmeriSpeak studies, NORC’s database is firewall protected and its data are encrypted. Only specific NORC personnel with appropriate clearance may access your confidential data.

[SPACE]

Our survey is anonymous. We are not asking for identifiable information. NORC will never give the research sponsors identifiable information. The results of our study may be published but your name will not be used. (In fact, the research sponsors will never know your name.)

[SPACE]

<u>PARTICIPATION AND WITHDRAWAL</u>

You can choose whether or not to be in this study. If you give permission but later decide that you do not want to participate in this study, you can exit the survey(s) and/or ignore the survey link(s). You will be compensated for the survey(s) that you complete.

[SPACE]

<u>IDENTIFICATION OF INVESTIGATORS</u>

If you have any questions or concerns about the research, please feel free to contact:

[SPACE]

Dr. Yalda T. Uhls

Department of Psychology, UCLA

1285 Franz Hall

310-210 1244 | yaldatuhls@gmail.com

[SPACE]

<u>RIGHTS OF RESEARCH SUBJECTS</u>

If you have questions about your rights as a research subject, or you have concerns or suggestions and you want to talk to someone other than the researchers, you may contact the UCLA Office of the Human Research Protection Program by phone: (310) 206-2040; by email: participants@research.ucla.edu or by mail: Box 951406, Los Angeles, CA 90095-1406

[SP]

CONSENT.

In the event that you disclose information about your possible intentions to do harm to yourself or others, NORC will provide information about your intentions to your parent(s) or legal guardian(s).

[SPACE]

Now that you know more about this study, you can make an informed decision about participation. Would you like to continue?

RESPONSE OPTIONS:

1. Yes
2. No
