# Supplementary material for: Investigating Viewership of Season 3 of “13 Reasons Why” and the Mental Wellness of Adolescents: Partially Randomized Preference Trial
Source: JMIR Ment Health. 2021 Sep 15;8(9):e25782. doi: 10.2196/25782 (PMC8482170; doi:10.2196/25782)
Supplement: Multimedia Appendix 3 [file mental_v8i9e25782_app3.docx]

**APPENDIX C. RESOURCES FOR PARENTS/GUARDIANS/HEADS OF HOUSEHOLD**

If you are currently having serious thoughts about suicide or feel you are in immediate danger, please dial 911 or go to your nearest emergency room. We also urge you to contact your primary healthcare provider or counselor, or you can find a licensed psychologist in your area by looking one up on https://locator.apa.org/.

Please also see these other resources that can help with feelings of distress or discomfort:

- **13reasonswhy.info**
- **stopbullying.gov**
- **crisistextline.org**
- **suicidepreventionlifeline.org**
- **rainn.org**
- **1-800-273-TALK (8255)**
- **Text HOME to 741741**
