# Supplementary material for: Investigating Viewership of Season 3 of “13 Reasons Why” and the Mental Wellness of Adolescents: Partially Randomized Preference Trial
Source: JMIR Ment Health. 2021 Sep 15;8(9):e25782. doi: 10.2196/25782 (PMC8482170; doi:10.2196/25782)
Supplement: Multimedia Appendix 4 [file mental_v8i9e25782_app4.docx]

**Appendix D POST SURVEY INTERVENTION**

**QS1. How much *13 Reasons Why* have you watched?**

*Please select all that apply*.

RESPONSE OPTIONS:

1. Some of Season 1 (2-5 episodes)

2. Most of Season 1 (6-10 episodes)

3. All of Season 1 (all 13 episodes – 13 episode with portrayal of actual suicide)

4. All of Season 1 (all 13 episodes – edited 13th episode without portrayal of actual suicide)

5. Some of Season 2 (2-5 episodes)

6. Most of Season 2 (6-10 episodes)

7. All of Season 2 (all 13 episodes)

8. Some of Season 3 (2-5 episodes)

9. Most of Season 3 (6-10 episodes)

10. All of Season 3 (all 13 episodes)

**Q1. How often have you spoken about the following with your friends in the last 30 days?**

RANDOMIZE GRID ITEMS:

A. Suicide (considering and attempting)

B. Mental health (for example, severe anxiety, anorexia, depression, etc.)

C. Bullying (online and offline)

D. Sexual assault (for example, inappropriate touching, lewd comments, rape, etc.)

E. Rigid gender stereotypes (for example, boys will be boys, girls are too emotional, etc.)

F. Substance abuse (drugs, alcohol, etc.)

G. Sexuality (for example, hookups, gender identity, sexual orientation, etc.)

RESPONSE OPTIONS:

1. Not at all

2. Once a week

3. A few times a week

4. Every day or almost every day of the week

**Q2. How strongly do you agree with each of the following statements?**

RANDOMIZE GRID ITEMS:

A. I can recognize my own mental health-related “warning signs,” or indicators that my own mental health may be poor.

B. I know how to find helpful mental health-related information and/or professional support.

C. If/whenever I need mental health-related information and/or professional support, I would reach out to get what I need.

D. If I felt suicidal, I would “tough” it out on my own rather than seek help.

RESPONSE OPTIONS:

1. Strongly disagree

2. Disagree

3. Neither agree nor disagree

4. Agree

5. Strongly agree

**Q3. Choose how many times you did the following (online or offline) in the last 30 days.**

RANDOMIZE GRID ITEMS:

A. I called other students names.

B. I said things about students to make other students laugh.

C. I threatened to hit or hurt another student.

D. I reached out to someone to apologize for how I’d treated them.

RESPONSE OPTIONS:

1. Never

2. 1 or 2 times

3. 3 or 4 times

4. 5 or more times

**Q4. For each of the following questions, choose how many times these things happened to you in the last 30 days.**

RANDOMIZE GRID ITEMS:

A. Other students made fun of me (online or offline)

B. I got hit and pushed by other students

C. Other students called me names

RESPONSE OPTIONS:

1. Never

2. 1 or 2 times

3. 3 or 4 times

4. 5 or 6 times

5. 7 or more times

**Q5. Indicate the likelihood you would be willing to engage in each behavior:**

RANDOMIZE GRID ITEMS:

A. Speak up if somebody made a threatening remark to another person

B. Ask someone who looks very upset if they’re okay or need help

C. Tell someone that you feel concerned about their unsafe choices

D. Post something on social media that supports someone who is being bullied

RESPONSE OPTIONS:

1. Not at all

2. Somewhat likely

3. Very likely

4. Extremely likely

**Q6. For each statement, please indicate how often this occurs:**

RANDOMIZE GRID ITEMS:

A. I feel sorry for someone who is treated unfairly.

B. I can easily tell how others are feeling.

C. I try to understand what other people go through.

RESPONSE OPTIONS:

1. Never

2. Rarely

3. Sometimes

4. Often

5. Always

**Q7. For each statement, please tell us if this has occurred:**

RANDOMIZE GRID ITEMS:

A. Have you ever been forced to have sexual intercourse when you did not want to?

B. Have you ever been forced to do sexual things that you did not want to do? (Count such things as kissing, touching)

C. Are you close with anyone (friend, family member, romantic or sexual partner) who has ever forced someone else to do sexual things that the other person did not want to?

RESPONSE OPTIONS:

1. Yes

2. No

77. Don’t know

**Q8. How much do you agree or disagree with the following?**

RANDOMIZE GRID ITEMS:

A. I know how to tell if my partner is ready to be physically intimate

B. I feel pressure from parents, teachers, or society to be physically strong

C. I feel pressure to dominate or be in charge of others

D. I feel pressure to hide my feelings when I am sad and/or anxious

E. I feel pressure to join in when my friends talk about others in a sexual way

RESPONSE OPTIONS:

1. Strongly disagree

2. Disagree

3. Neither agree nor disagree

4. Agree

5. Strongly agree

**Q9. Have you ever had thoughts of suicide?**

RESPONSE OPTIONS:

1. Never

2. Once

3. A few times

**Q10. How much do you agree or disagree with the following statements?**

RANDOMIZE GRID ITEMS:

A. At times, I think I am no good at all

B. I certainly feel useless at times

C. I wish I could have more respect for myself

RESPONSE OPTIONS:

1. Strongly disagree

2. Disagree

3. Neither agree nor disagree

4. Agree

5. Strongly agree

**Q11. How much do you agree or disagree with the following statements?**

RANDOMIZE GRID ITEMS:

A. A lot of adolescents experience depression

B. It’s hard to tell if someone you know abuses or is addicted to drugs, alcohol, etc.

C. There are not enough resources to help teens deal with suicide

D. Most people who commit sexual assault receive formal punishment that’s as serious as the crime

E. “Acting like a man” today means to act tough, to hide emotions and to pursue women

F. When women publicly speak out against sexual abuse, they tend to get harassed

G. Most teens who reach out for support around mental health are glad that they did

H. People whose orientation is anything other than straight (e.g., homosexual, bisexual) have to hide their sexuality to be socially accepted

RESPONSE OPTIONS:

1. Strongly disagree

2. Disagree

3. Neither agree nor disagree

4. Agree

5. Strongly agree

**Q12. How often have you been bothered by each of the following symptoms during the past 7 days?**

RANDOMIZE GRID ITEMS:

A. Feeling down, depressed, irritable, or hopeless

B. Little interest or pleasure in doing things

C. Trouble falling asleep, staying asleep, or sleeping too much

RESPONSE OPTIONS:

1. Not at all

2. Several days

3. More than half of the days

4. Nearly every day

**Q12A. Was it hard to not watch *13 Reasons Why* Season 3?**

RESPONSE OPTIONS:

1. Yes

2. No

3. Other, please specify: [TEXTBOX]

**Q12B. Did you see any of the following in the last month?**

RANDOMIZE GRID ITEMS:

A. Press (articles, news items) on 13 Reasons Why

B. Social Media about 13 Reasons Why

C. Trailers (promotional videos from Netflix) about 13 Reasons Why

D. Reviews on 13 Reasons Why

RESPONSE OPTIONS

1. Not at all

2. Several

3. More than half the days

4. Nearly every day

**Q13. With whom did you discuss *13 Reasons Why* and issues related to the show after watching?**

*Please select all that apply*.

RESPONSE OPTIONS:

1. Friends

2. Parents

3. Partner, boyfriend or girlfriend

4. A sibling

5. Other family members

6. Teacher

7. School counselor

8. Mental health professional or someone at a mental health resource hotline

9. I did not discuss the show with anyone [SP]

10. Other, please specify: [TEXTBOX]

**Q14. Please tell us a bit more. Which part of the show did you discuss, what did you talk about and was it helpful? Please give us as much detail as you can.**

**Q15. Which social media profiles affiliated with *13 Reasons Why* did you visit while watching the show?**

*Please select all that apply*.

RESPONSE OPTIONS:

1. Instagram Profiles

2. Twitter

3. Facebook

4. Other

5. None [SP]

**Q15A. Please specify each social media profile you visited while watching *13 Reasons Why* below.**

*Please select all that apply*.

Instagram Profiles

1. @13ReasonsWhy

2. @dylanminnette

3. @christianleenavarro

4. @AlishaBoe

5. @flynnagin11

6. @Justin.Prentice

7. @younggoth

8. @RossButler

9. @katherinelangford

10. Other, please specify: [TEXTBOX]

Twitter

11. @13ReasonsWhy

12. @dylanminnette

13. @ChristianLN0821

14. @AlishaBoe

15. @brandonflynn

16. Other, please specify: [TEXTBOX][JM1]

**Q16. Which crisis/informational resources did you visit or watch that are affiliated with the show?**

*Please select all that apply*.

RESPONSE OPTIONS:

1. www.13reasonswhytoolkit.org

2. 13reasonswhy.info

3. Discussion Guide on the *13 Reasons Why* info web page

4. Beyond The Reasons video on Netflix

5. I didn’t visit any crisis/ information resources [SP]

6. Other, please specify: [TEXTBOX]

**Q17. My favorite character on the show was (choose one). Click on character name for reminder of who they are.**

RESPONSE OPTIONS:

1. Ani Achola
2. Alex Standall
3. Bryce Walker
4. Caleb
5. Charlie
6. Chloe Rice
7. Clay Jensen
8. Hannah Baker
9. Jessica Davis
10. Justin Foley
11. Montgomery de la Cruz
12. Tyler Down
13. Other, please specify: [TEXTBOX]

**Q18. How much do you agree or disagree with the following?**

GRID ITEMS:

1. [INSERT Q17 RESPONSE] reminds me of myself
2. I have the same problems as [INSERT Q17 RESPONSE]
3. I care about what happens to [INSERT Q17 RESPONSE]
4. While watching the show, I could feel the emotions that [INSERT Q17 RESPONSE] felt
5. Throughout the show, I felt I knew exactly what [INSERT Q17 RESPONSE] was going through

RESPONSE OPTIONS:

1. Strongly disagree

2. Disagree

3. Neither agree nor disagree

4. Agree

5. Strongly agree

**Q19. How much do you agree or disagree with the following?**

GRID ITEMS:

1. I could picture myself in the scene of the events depicted in the show.
2. I was mentally involved in the show while watching it.
3. I wanted to learn how the show ended.
4. The show affected me emotionally.
5. While viewing *13 Reasons Why*, I forgot myself and was fully absorbed

RESPONSE OPTIONS:

1. Strongly disagree

2. Disagree

3. Neither agree nor disagree

4. Agree

5. Strongly agree

**Q20. The third season of *13 Reasons Why* portrayed teen life…**

RESPONSE OPTIONS:

1. Completely unrealistically
2. Somewhat unrealistically
3. Realistically
4. Totally realistically
5. It is not like my life, but it is like most other teen’s lives

**Q21. During or after watching the show, due to something you watched in the show, did you try to learn more about any of the following (through online search, asking an adult, etc.)?**

*Please select all that apply*.

RESPONSE OPTIONS:

1. Suicide (considering and attempting)
2. Mental health (for example, severe anxiety, anorexia, depression, etc.)
3. Bullying (online and offline)
4. Sexual assault (for example, inappropriate touching, lewd comments, rape, etc.)
5. Rigid gender stereotypes (for example, boys will be boys, girls are too emotional, etc.)
6. Substance abuse (for example, drugs, alcohol, etc.)
7. Sexuality (for example, hookups, gender identity, sexual orientation, etc.)
8. Abortions
9. Illegal immigration
10. Other, please specify: [TEXTBOX]

**Q22. How often did you watch *13 Reasons Why*…**

GRID ITEMS:

1. ...alone?
2. ...using headphones?
3. ...with a parent?
4. ...with a friend/sibling?
5. ...in binge fashion (one episode right after another)?
6. ...after 9 PM?

RESPONSE OPTIONS:

1. Never

2. Rarely

3. Sometimes

4. Very often

5. All of the time

**Q23. Who accompanies Chloe to get her abortion?**

RESPONSE OPTIONS:

1. Clay Jensen

2. Bryce Walker

3. Zach Dempsey

4. Jessica Davis

5. No one. She goes alone.

**Q24. Who tipped off ICE about Tony’s family?**

RESPONSE OPTIONS:

1. Caleb (Tony’s boyfriend)

2. Mr. Walker (Bryce’s Father)

3. Bryce Walker

4. Montgomery de la Cruz

5. Tony’s neighbor

**Q25. Who killed Bryce Walker?**

RESPONSE OPTIONS:

1. Alex Standall

2. Zach Dempsey

3. Clay Jensen

4. Jessica Davis

5. Ani Achola

**Q26. It looks like you may not have paid close attention to the show or stopped watching. That’s ok, please help us by answering which of the following is true for you:**

RESPONSE OPTIONS:

1. I stopped watching
2. I watched, but I guess I didn’t pay close attention

**Q26A. Please tell us at what episode you stopped watching and why.**

**Q27. How do you feel the show impacted you? Is there anything else you would like to add?**
